# Keeping Our Eyes on the Prize: Focusing on Parenting Supports Depressed Parents’ Involvement in Home Visiting Services

**DOI:** 10.1007/s10995-018-2533-y

**Published:** 2018-05-28

**Authors:** Lorraine M. McKelvey, Shalese Fitzgerald, Nicola A. Conners Edge, Leanne Whiteside-Mansell

**Affiliations:** Department of Family and Preventive Medicine, College of Medicine, University of Arkansas for Medical Sciences, 4301 W. Markham St, #530, Little Rock, AR 72205-7199 USA

**Keywords:** Home visiting, Depression, Retention, Family engagement

## Abstract

*Objectives* Improving family retention and engagement is crucial to the success of home visiting programs. Little is known about retaining and engaging depressed parents in services. The purpose of the study is to examine how home visit content moderates the association between depression and retention and engagement. *Methods* The sample (N = 1322) was served by Healthy Families America (n = 618) and Parents as Teachers (n = 704) between April 1, 2012 and June 30, 2016. Parents averaged 23 years (SD = 6). Nearly half of the parents were White (48%) and the majority was single (71%). Depression was screened with the Patient Health Questionnaire-2. Home visitors reported the percent of time focused on particular content and parent engagement at every home visit. *Results* Multilevel regression analyses showed the amount of time that home visitors spent supporting parent–child interaction moderated the association between depression and retention at 6 (*B* = .08, *SE* = .03, *p* = .003) and 12 (*B* = .1, *SE* = .03, *p* < .001) months, such that there was a stronger positive association for depressed parents. The main effects of child development focused content and retention at 6 (*B* = .07, *SE* = .01, *p* < .001) and 12 (*B* = .08, *SE* = .01, *p* < .001) months were positive, while effects of case management focused content at 6 (*B* = − .06, *SE* = .01, *p* < .001) and 12 (*B* = − .07, *SE* = .01, *p* < .001) months were negative. *Conclusions* Families were more likely to be retained when home visitors focused on child development and parent–child interaction, but less likely with more case management focus. Parents with positive depression screens were more likely to remain in services with more time spent focused on supporting parent–child interactions.

## Significance

Depressive symptoms are common among parents receiving home visiting services and may impede the effectiveness of the intervention. Our findings provide guidance to home visitors, indicating that they may increase the likelihood that parents will remain in the intervention if they stay focused on supporting the parent–child relationship. This is especially true for parents with depressive symptoms.

## Introduction

Parental depression has significant negative impacts on children’s development. A meta-analysis of 46 studies (Lovejoy et al. 2000) reported that depression interferes with the parent’s capacity to provide engaged and positive caregiving. Specifically, maternal depression significantly increased the likelihood of interactions where maternal behaviors with children were negative in affect (i.e., irritable, hostile or coercive) and were less emotionally available, reciprocal, and positive in interactions with their children. Findings from the study are especially salient during infancy. Further, the effects of depression in infancy are evident even when depressive symptoms do not meet the criteria for a major depressive disorder (Conners-Burrow et al. 2014, 2015, 2016).

Home visiting interventions provide one avenue to improve child outcomes in families where children are at risk, including when parents are depressed. The theory of change for many evidence-based home visiting (EBHV) interventions is to promote the optimal growth and development of children through enhancing the quality of parenting and family environments (Raikes et al. 2014). While most EBHV programs do not purposefully recruit parents with depression [notable exceptions include In-Home Cognitive Behavior Therapy through Moving Beyond Depression™; Ammerman et al. 2015 and Enhanced Triple P, (Sanders et al. 2000)], serving parents with depression is common. Results from the national study of home visiting (Mother and Infant Home Visiting Program Evaluation) suggest that 40% of mothers exhibited depression or anxiety at enrollment (MDRC 2017). Indeed, screening for depression is required when programs receive Maternal, Infant, and Early Childhood Home Visiting (MIECHV) funding. It is clear that caregivers with depression enroll in EBHV; however, what is known about their participation and persistence in programs is variable (Azzi-Lessing 2013; Sweet and Appelbaum 2004). Several studies reported no association between caregiver depression and retention in services, including: SafeCare+ (Damashek et al. 2011), Durham Connects (Navaie-Waliser et al. 2000), Nurse-Family Partnership (Brand and Jungmann 2014; O’Brien et al. 2012), and Parenting for Life (Booth et al. 2014). Similarly, a study of a French perinatal home visiting program also reported no association between program attrition and caregiver depression at enrollment but found that caregivers with higher levels of general psychiatric symptoms were less likely to remain in services (Foulon et al. 2015). Positive associations between depressive symptoms and retention in home-visitation programs have also been reported in Family Connections (Girvin et al. 2007), and Healthy Families America and Nurse-Family Partnership (Ammerman et al. 2006, 2009).

Recent studies have explored associations between how the home visitor focuses the visit and engagement and retention. Home visitors may focus on facilitating parent–child interactions, (i.e., supporting reciprocity of interactions by helping the parent understand their child’s cues and respond with warmth and empathy). They may also deliver child-focused content, for example, sharing information about the child’s development. Other times home visitors feel obligated to address family needs and may engage in more case management like activities. In Early Head Start (EHS), the more time home visitors spent facilitating parent–child interactions (Peterson et al. 2007) and delivering child-focused content (Roggman et al. 2008), the greater parental engagement in services.

Participant engagement and attrition is a major influence on the effectiveness of home visiting programs (Azzi-Lessing 2011). It is clear that monitoring participant engagement and attrition, assessing family and program characteristics associated with attrition and engagement, and developing strategies to retain and engage at-risk families are crucial for the successful replication of EBHV programs (Azzi-Lessing 2011, 2013). The purpose of the current study is to investigate how program process variables—specifically the percent of time home visitors focus the content of home visits on parent–child interactions, child development, and parent/family case management—influence the association between caregiver depression at enrollment and engagement and retention in services. While we are the first study to investigate whether particular program content moderates the association between program engagement and depression at enrollment, we can extrapolate from existing studies of program content provided within the Early Head Start (EHS) model. EHS serves a similar population of families as the EBHV models included in this study. Those studies, which included smaller samples of families, suggest that remaining focused on child development and parent–child interaction should increase parental engagement in programs, regardless of depressive symptoms at enrollment.

## Methods

### Study Design

This study uses data collected during the implementation of EBHV programs funded through the MIECHV program in Arkansas. Families included in the analysis voluntarily enrolled in two EBHV models, Healthy Families America (HFA) and Parents as Teachers (PAT), which serve expectant families and those with children up to age three. The overall goal of the HFA program is to promote child well-being and prevent abuse and neglect. The PAT program, designed as a universal parenting intervention, focuses on the promotion of optimal early development, learning, and health. Families were eligible for services if they reported characteristics associated with less optimal parenting, including (1) demographic (low-income or a single and/or teen parent), parent (such as parental history of abuse, incarceration, military deployment, disability, or chronic illness), and child (developmental delay, pre-term/low birth weight, disability, or chronic illness) risks. All families provided consent to have their data used for evaluation purposes. The University of Arkansas for Medical Sciences Institutional Review Board approved the study.

### Participants

We used program process data collected about families who were enrolled for at least one month between April 1, 2012 and June 30, 2016 (N = 1322; HFA N = 618, PAT N = 704) to examine the retention and engagement patterns of parents who screened positive for depression. The majority of families (89%) were living at or below 100% of the federal poverty line. Primary caregivers were 23 years old on average (*SD* = 6). Nearly half were Caucasian (48%) with another 25% being African-American and 23% of Hispanic ethnicity. The majority of primary caregivers reported having education at or less than a high school or general equivalency diploma (78%) and were single (75%). Children were 4 months of age on average (*SD* = 10) and were equally divided on gender (50% male). Table 1 shows family and child demographics at enrollment.

**Table 1:** Sample demographics at enrollment

	Percentage (*n* = 1322)
Age of applicant in years (*M, SD*)	23 (6)
Teenage motherhood	
Mother < 20	38.5%
Mother 20 or older	61.5%
Applicant race/ethnicity	
Caucasian	48.1%
African-American	25.1%
Hispanic	22.7%
Other	4.1%
Applicant education	
Less than high school graduate	43.0%
High school graduate or equivalent	35.4%
Some college or degree	21.6%
Applicant employment status	
Unemployed	70.6%
Part time	14.0%
Full time	15.4%
Marital status	
Single	70.9%
Married/cohabiting	25.2%
Separated, divorced or widowed	3.9%
Family income (*M, SD*)	$9741 ($8108)
Poverty (100% or less)	89.0%
Number of adults in family (*M, SD*)	1.6 (1.2)
Number of children in family (*M, SD*)	1.6 (1.3)
Prenatal enrollment	46.0%
Child age at enrollment in months (*M, SD*)	4 (10)
Child is male	50.4%
Parental depression risk (scores) at enrollment	
Not at risk (0–1)	81.6%
At risk (2 or higher)	18.4%

### Measures

*The Family Map Inventories* (FMI; Whiteside-Mansell et al. 2007, 2013) were completed within 1 month of enrollment. The FMI are a set of semi-structured interviews developed to assess important aspects of the family and home environment associated with child well-being. There are three versions of the FMI based on the age of the child at the time of assessment: Prenatal, Infant/Toddler, and Early Childhood. The instruments, designed for use during home visits, systematically identify areas of strength and concern associated with healthy child development. All versions of the FMI include the *Patient Health Questionnaire-2* (Kroenke et al. 2003) screening for depression. The two-item PHQ-2 is efficient, well validated and recommended by the U.S. Preventive Services Task Force as a good screening option for depressive symptoms (Pignone et al. 2002). Response options on the PHQ-2 include ‘not at all’ (0), ‘several days’ (1), ‘more than half the days’ (2), and ‘nearly every day’ (3). Sum scores on the PHQ-2 range from 0 to 6, with higher scores representing a greater endorsement of depressive symptoms.

There are multiple recommendations for determining the cutoff of the instrument for predicting depression. In the original development study, a cutoff score of 3 was reported to have a sensitivity of 83% and a specificity of 92% for predicting major depressive disorders, while a cutoff of 2 increased the sensitivity to 93% (Kroenke et al. 2003). A recent study of a primary care population (Arroll et al. 2010) demonstrated sensitivity and specificity of the PHQ-2 for diagnosing major depression of 86 and 78% with a score of 2 or higher, and 61 and 92% with a score 3 or higher. As sensitivity of the tool increased 25% using a lower threshold and there were 63 patients with major depressive disorder that were undetected using the higher threshold, the authors concluded that individuals scoring 2 or higher should be further screened in clinical settings. There are also multiple studies that document the impact of lower-level depression on parenting in low-income samples (Conners-Burrow et al. 2014, 2015, 2016). As a result, we examine parents scoring at 2 or higher.

Home visitors documented each home visit. Program staff entered individual services into a web-based data management system, Efforts to Outcomes (ETO; Social Solutions 2016), designed to track all elements of home visiting programs, including family contacts with and without educational content, unsuccessful visits (e.g., the home visitor arrives to find no one is home), and dismissals. Home visits were documented in ETO using a Home Visit Record (HVR). The HVR was developed by modifying the Home Visit Contents and Characteristics observation record of the Baby FACES study (Vogel et al. 2011). In brief, the HVR includes the home visitor (N = 96) reports of the percentage of the time of the total home visit that was focused on specific content (parent-child-focused, child-focused, and parent/family-focused), and home visitor ratings of parent engagement. Home visitors reported the percent of time focused on particular content and parent engagement at every home visit. The evaluation team trained home visitors on all measures. Further, to assure that home visitors were clear of the intent of the reporting, the evaluation team built the data system such that the HVR included the definitions described below.

We aggregated and averaged the percentages across home visits. *Parent–child interaction* content was defined for staff as “focused on the parent–child dyad, for example, activities to enhance parent–child interactions or the parent–child relationship” (*M* = 16.99, *SD* = 12.17). *Child development* content “focused on the child and his/her development, for example, activities with the child to promote child development, child development assessment and provision of parenting education on developmental milestones, etc.” (*M* = 26.85, *SD* = 19.24). Parent/family content was defined as “case management, family support, and adult education on other topics” (*M* = 30.41, *SD* = 16.87).

Program process outcomes examined in the current study include retention and engagement in services. There are multiple reasons why families leave services. The models included in this analysis have differing policies for discharging families. Parents as Teachers does not have a national policy and allows programs to make decisions on a case-by-case basis. Healthy Families America has a service level called “Creative Outreach” where families with whom home visitors have lost contact can remain on the caseload for 90 days as the program works to re-establish communication, but it is not required that the program use the full amount of time before replacing the client. Therefore, there is a great deal of variability in when families are discharged from services, across models, programs, and families. For all process outcomes in this study, families’ dismissal dates were set to the date of their last successfully completed home visit. *Family retention* represents a dichotomy (0 = No, 1 = Yes) of remaining in the program a minimum of 6 (71% retention rate) and 12 (48% retention rate) months.

We use two measures of family engagement. We computed a *home visit completion* ratio, computed as the total number of completed home visits to all attempted and completed visits. Higher scores on home visit completion represent a family being accessible for home visits at a higher rate (*M* = 0.80, *SD* = 0.17). Since families who often ‘no show’ are less engaged in services (Ingoldsby 2010), this variable represents an objective measure of engagement. The HVR includes home visitor ratings of parent engagement. Home visitors were provided instructions for rating engagement: “Indications of engagement in the activity include: (1) asking questions about materials, (2) asking questions about applications of the topic, (3) seeing the parent apply the concepts discussed, and (4) hearing/seeing the mother talk to other family members about materials concepts discussed.” *Engagement* was rated on the scale: (1) Less than 10%, (2) 10–24%, (3) 25–50%, (4) 51–75%, (5) 76–90%, and (6) Over 90%. Ratings were aggregated and averaged across home visits, and the mean reflects a high endorsement of engagement (*M* = 5.48, *SD* = 0.5).

### Approach to Analysis

Because the data are nested within program model and home visitor, we computed intraclass correlations (ICCs) to determine whether generalized linear modeling techniques are appropriate. While there is not a clear consensus on the strength of ICCs that require employing multilevel models (MLM), the ICCs for home visitor accounted for more than 5% of the variance in the engagement and retention outcomes, so they were modeled (Raykov 2011). MLM (Raudenbush and Bryk 2002) refers to a class of statistical techniques developed to analyze and appropriately model clustered designs. This analysis allowed us to explicitly model the variance in engagement and retention accounted for by the home visitor-level effects. Models were fit using R (Bates et al. 2014; R Core Team 2015; Zeileis and Hothorn 2002). We probed significant interaction terms in post hoc simple slope analyses, which investigate the relationship one standard deviation above and below the mean for continuous predictors (Aiken et al. 1991; Dawson and Richter 2006; Preacher et al. 2006). Significant interaction terms indicate that the slopes of the two lines are different from each other; however, it is possible that regression slopes not be statistically different from zero (Preacher et al. 2006).

We used MLM to examine the moderating role of home visiting content on the association between depressive symptoms at enrollment (0 = Negative Screening, 1 = Positive Screening) and the program process outcomes described above. Models included the fixed effects of parental age, race, education, employment, and marital status, the number of adults and children in the home, and child age, as well as the random effect of home visitor and home visiting model. Models included the main effects of depressive symptoms at enrollment, home visiting content, and their interaction. When interaction terms were not significant, we reported the main effects of depression and home visiting content.

Missing data consisted of both invalid assessments and missing values. Assessments given outside the enrollment window were dropped from analysis (9.8%). Missing values for variables in the data set were relatively low, ranging from 0.4 to 8.7%. There is some assertion in the literature that small amounts of missing data, 5% (Schafer 1999) to 10% (Bennett 2001), are inconsequential. Indeed, a recent study of procedures for handling missing data demonstrated that under the missing at random condition that model estimates were unbiased even in the case of 20% missing data (Dong and Peng 2013). Because of the small amount of missingness and computational limitations of imputing unbiased categorical estimates in hierarchical data, we analyzed complete cases.

## Results

### Family Retention

**Fig. 1:**
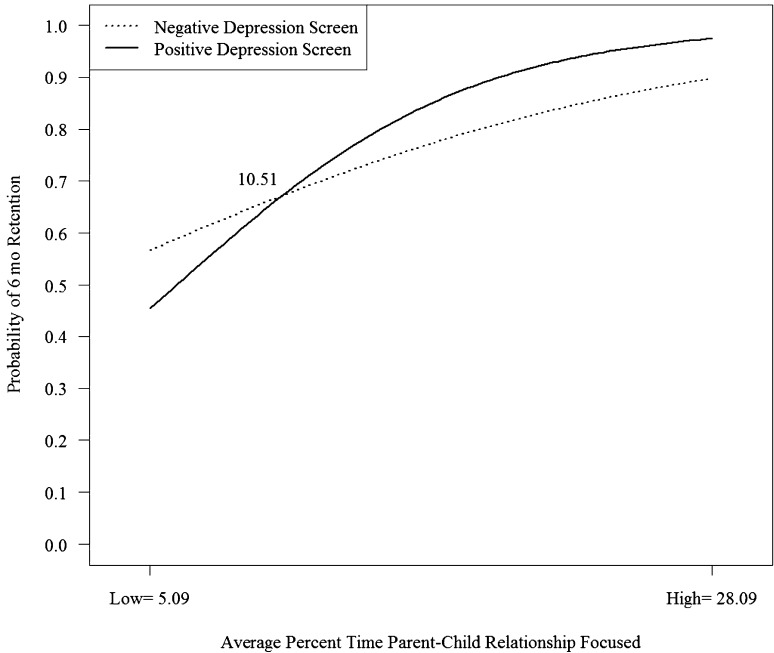
Interaction of depression screening at enrollment and home visiting time spent focused on supporting the parent–child relationship on retention in services at 6 months

**Fig. 2:**
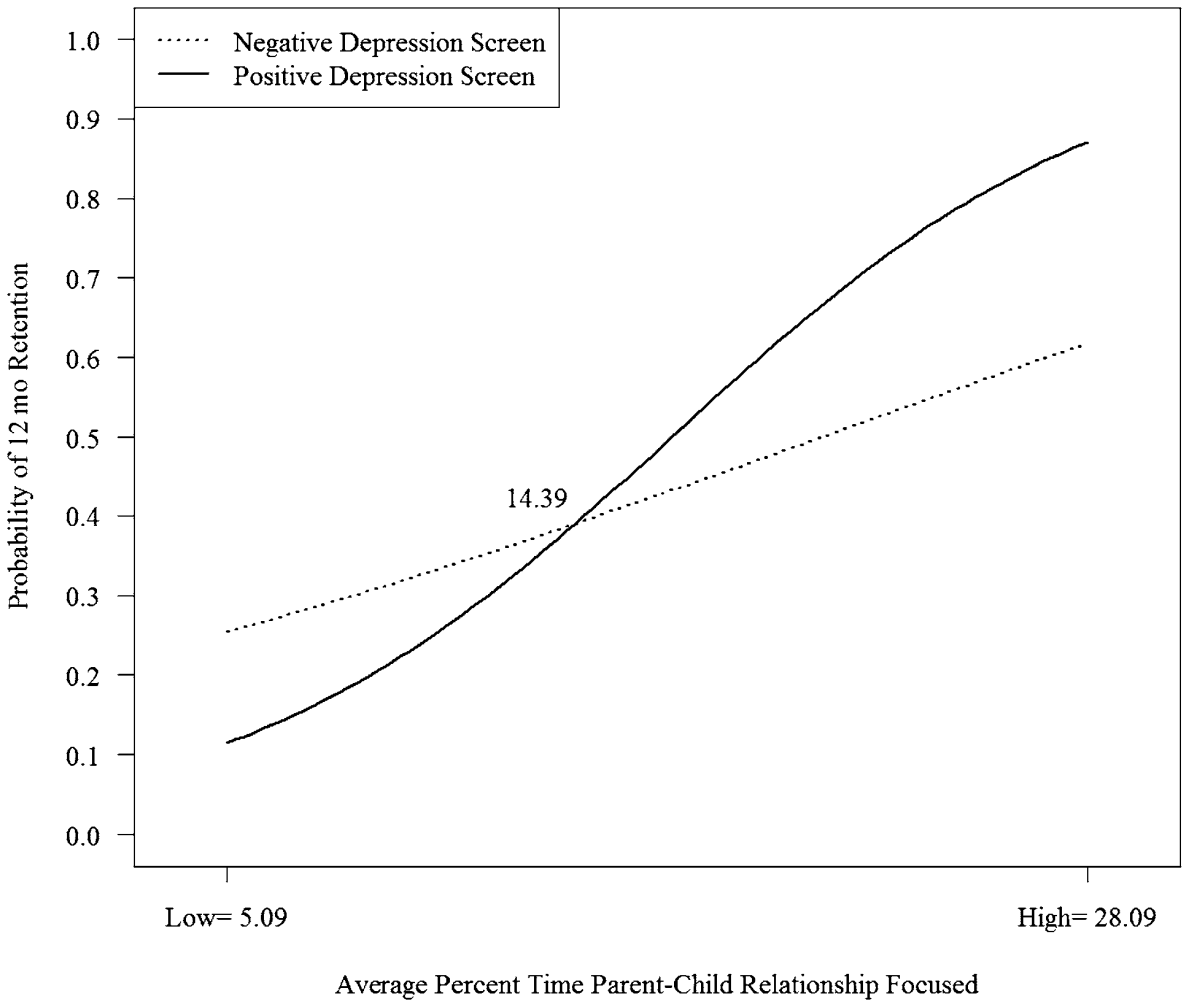
Interaction of depression screening at enrollment and home visiting time spent focused on supporting the parent–child relationship on retention in services at 12 months

The two-way interaction with the moderator parent–child interaction was significant for predicting retention at 6 (*B* = .08, *SE* = .03, *p* = .003) and 12 (*B* = .1, *SE* = .03, *p* = .000) months (see Table 2). Simple slope analyses reveal that a greater amount of time focused on the parent–child relationship is associated with a higher probability of remaining in services at 6 months (Fig. 1) and 12 months (Fig. 2) and that there is a stronger association for parents with higher than lower depressive symptoms at both retention endpoints. The point of intersection (i.e., the point at which the relationship between parent–child interaction content and retention becomes stronger for depressed versus non-depressed families) was 11% of time across all home visits where content is parent–child interaction focused for retention at 6 months and about 14% for 12 months. This represents a relatively small percentage of overall home visit time. As the average amount of time spent on parent–child interaction content across all home visits was approximately 17% (*SD* = 12%), the intersection represents one-half a standard deviation below the average for retention at 6 months and one-quarter a standard deviation below the average for retention at 12 months.

**Table 2:** Path coefficients of depression screening at enrollment and parent–child relationship focused home visiting content and predicting service retention and engagement

Construct	Retention at 6 months	Retention at 12 months	Engagement: home visit completion
	B (SE)	B (SE)	B (SE)
Covariates			
Parent age at enrollment (years)	− 0.03 (0.02)	− 0.02 (0.02)	− 0.001 (0.001)
Child age at enrollment (months)	− 0.01 (0.01)	− 0.02* (0.02)	− 0.000 (0.001)
Employment	− 0.13 (0.14)	− 0.04 (0.12)	− 0.011† (0.007)
Parent education	0.22** (0.08)	0.18** (0.07)	0.003 (0.004)
Parent race/ethnicity: Black/African American	− 0.02 (0.31)	0.04 (0.29)	− 0.006 (0.018)
Parent race/ethnicity: Native American	− 2.16 (1.62)	− 0.25 (1.43)	0.002 (0.1)
Parent race/ethnicity: Asian	0.22 (0.96)	− 0.69 (0.63)	− 0.009 (0.05)
Parent race/ethnicity: Pacific Islander	1.28 (1.28)	1.72† (0.96)	0.04 (0.05)
Parent race/ethnicity: Multi-racial	1.32 (1.23)	0.2 (0.78)	− 0.025 (0.05)
Parent race/ethnicity: Hispanic/Latino	0.77* (0.33)	0.73* (0.29)	0.01 (0.017)
Marital status: married/co-habitation/domestic partnership	0.37 (0.27)	0.31 (0.21)	0.03* (0.013)
Marital status: separated	− 0.12 (0.72)	0.16 (0.65)	0.02 (0.05)
Marital status: divorced	0.45 (0.99)	1.39 (0.88)	0.03 (0.05)
Marital status: widowed	− 1.46 (1.56)	− 0.08 (1.5)	0.04 (0.136)
Number of minors in the home	− 0.07 (0.082)	− 0.02 (0.07)	− 0.006 (0.005)
Number of adults in the home	− 0.17† (0.09)	− 0.19* (0.08)	− 0.007† (0.005)
Model (0 = HFA, 1 = PAT)	0.05 (0.47)	0.02 (0.42)	0.004 (0.05)
Depression at enrollment (0 = No Risk, 1 = Risk)	− 0.88* (0.42)	− 1.5*** (0.42)	− 0.05* (0.03)
Parent–child relationship focus	0.08*** (0.02)	0.07*** (0.01)	0.004*** (0.001)
Depression X parent–child focus	0.08** (0.03)	0.1*** (0.03)	0.005*** (0.001)
Constant (SE)	0.16 (0.58)	− 1.17* (0.52)	0.78*** (0.04)

†*p* < .10, *p < .05, **p < .01, ***p < .001

We computed the regions of significance for the interaction, which indicates the value of the moderator (the percentage of time spent focused on parent–child interaction) at which association between depression and retention is significant. For 6 month retention, the lower bound of the region of significance was 9.14 (*B* = − .08, SE = 0.04, p = .05), which is approximately two-thirds of a standard deviation below the mean of time spent focused on parent child interaction. The slope is negative, which indicates that parents who screen positive for depression are significantly more likely to leave services than those who do not when less than average support of the parent–child relationship is provided. The upper bound of the region of significance was 22.01 (*B* = .08, SE = 0.04, p = .05), which is a little over one-third of a standard deviation above the mean of time spent focused on parent child interaction. The slope is positive, which indicates that parents who screen positive for depression are significantly less likely to leave services than those who do not when at more than average support of the parent–child relationship is the focus. For retention at 12 months, the lower bound of the region of significance was 12.17 (*B* = − .08, SE = 0.04, p = .05) and the upper bound was 21.99 (*B* = .09, SE = 0.05, p = .05).

The other areas of program focus, child development and parent/family (case management) did not moderate the association between depression and retention; as such, we interpret their main effects. The main effects of home visitors staying focused on child development had a positive association with retention at both 6 (*B* = .07, *SE* = .01, *p* < .001) and 12 (*B* = .08, *SE* = .01, *p* < .001) months. Therefore, the more time home visitors spent focused on child development, the more likely families were to stay in services, regardless of parent depression at enrollment. Home visitors staying focused on parent/family had a negative association with retention at 6 (*B* = − .06, *SE* = .01, *p* < .001) and 12 (*B* = − .07, *SE* = .01, *p* < .001) months. This finding suggests the more time home visitors spent focused on case management, the more likely families were to leave services.

### Family Engagement: Home Visit Completion

**Fig. 3:**
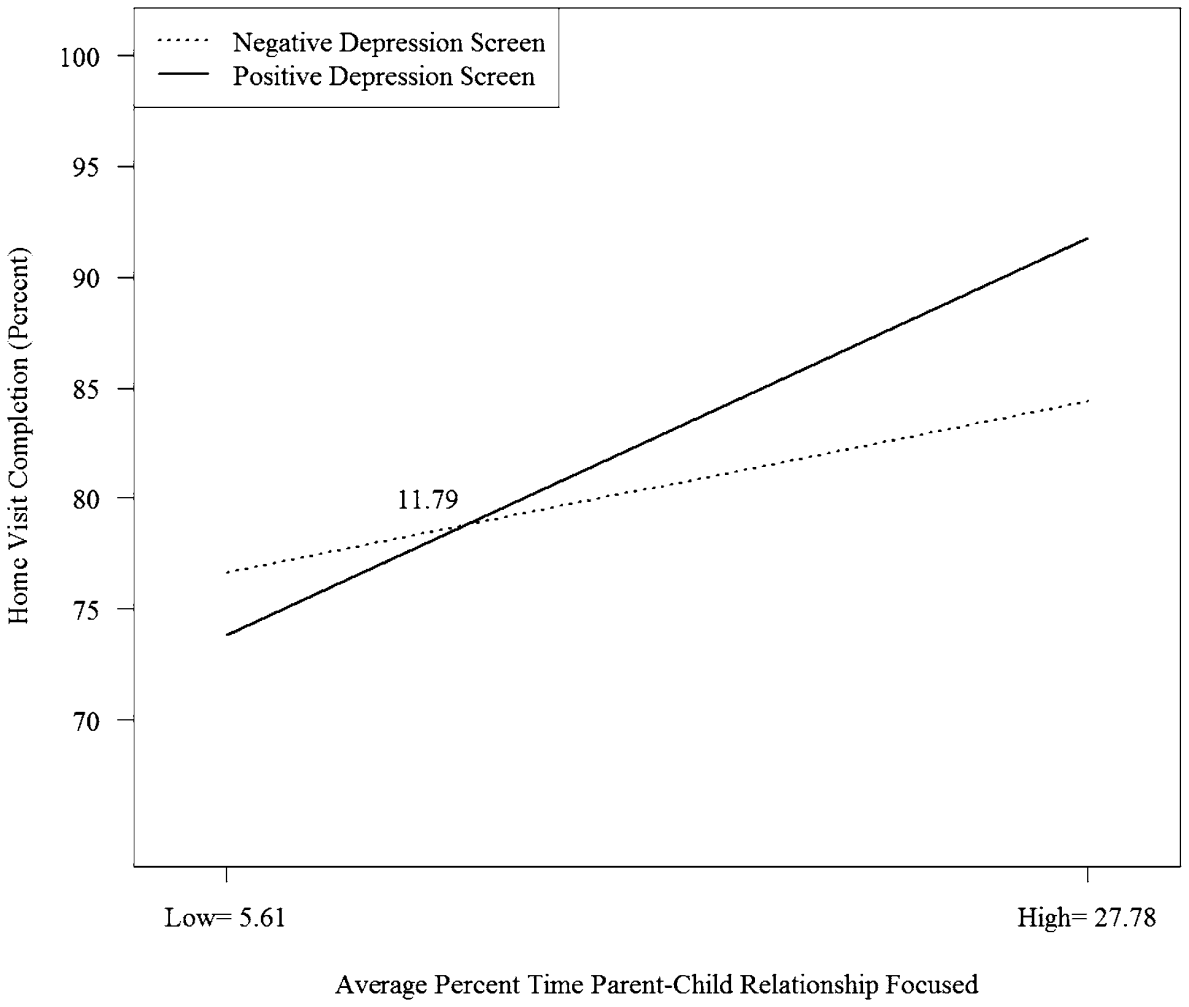
Interaction of depression screening at enrollment and home visiting time spent focused on supporting the parent–child relationship on home visit completion

The two-way interaction with the moderator parent–child interaction was significant for predicting home visit completion (*B* = .005, *SE* = .001, *p* < .001). Simple slope analyses for this interaction (Fig. 3) reveal a positive association between focus on supporting the parent–child relationship and successfully completed home visits, which is stronger for families with higher versus lower depressive symptoms. The point at which the relationship becomes stronger for depressed versus non-depressed families is relatively low, about 12% of the time across all home visits (i.e., slightly more than one-thirds a standard deviation below the average support for parent–child interaction across all families). The lower bound of the region of significance for home visit completion was 5.73 (*B* = − .04, *SE* = 0.02, *p* = .05), which is approximately one standard deviation below the mean of time spent focused on parent child interaction. The upper bound of the region of significance was 24.74 (*B* = .04, *SE* = 0.02, *p* = .05), which is a little over half of a standard deviation above the mean of time spent focused on parent child interaction.

As demonstrated with retention, focus on child development or parent/family (case management), did not moderate the association between depression and home visit completion. Focus on child development had a positive association with home visit completion (*B* = .002, *SE* = .001, *p* < .001). The more time home visitors spent focused on child development and parenting to support child development, the more likely families were to complete scheduled home visits. The home visit completion engagement variable was also significantly negatively associated with the time spent parent/family focused (*B* = − .004, *SE* = .001, *p* < .001). The more time home visitors spent focused on case management, the less likely families were to complete scheduled visits.

### Family Engagement: Home Visitor Ratings

There was no significant moderation of program content on the association between parental depression and home visitor ratings of engagement. The main effect of depression on reported engagement was negative and significant in the models containing parent–child interaction (*B* = − .095, *SE* = .045, *p* = .044), child development (*B* = − .07, *SE* = .042, *p* = .049), and parent/family case management (*B* = − .11, *SE* = .065, *p* = .044). Therefore, home visitors rated parents entering services with depressive symptoms as less engaged over the course of their services. Main effects of program content suggest that home visitors rate families as more engaged if they spend more time focused on parent/family case management (*B* = .002, *SE* = .001, *p* = .03). The associations between time spent on parent–child interaction, time spent on child development, and home visitor rating of engagement were non-significant.

## Discussion

Home visiting programs are well suited to support parents with depressive symptoms, such as lack of energy and diminished interest or motivation. Home visiting can reduce these symptoms by improving parent–child interactions and children’s development (Chazan-Cohen et al. 2007). However, we know relatively little about what is helpful in retaining and engaging depressed parents in EBHV. The current study examined the moderating role of content provided to families and the association between retention and engagement of depressed mothers in services.

Home visitors can feel pulled in many directions. They must balance the goals of the visit with their desire to meet the family’s concrete needs or to spend time nurturing their relationship with family members (Jones Harden et al. 2010). Our findings suggest that all families are more likely to complete visits and to remain in the program when home visitors devote sessions to child development and parent–child interactions. Further, parents with depressive symptoms are enrolled longer and are more engaged when home visitors focus on parent–child interactions.

For all families, there was a negative association between retention and visit completion and having more of a parent/family (or case management) focus in our services. That association remained even when we controlled for family risks, such as low education or unemployment, that might increase family need for case management. Theoretically, the individualization of services will produce greater parental involvement in home visiting (McCurdy and Daro 2001). Unfortunately, there is some evidence that family’s needs may not match the case management services they receive. For example, Tandon et al. (2005) reported over half of mothers participating in HFA experienced domestic violence, substance abuse, and/or a mental health diagnosis. According to HFA service documentation, only one-quarter of those mothers received services related to those risks. There is also data that supports that, in the absence of a home visitor, that families will find ways to meet their referral needs. Findings from one randomized trial of EHS reported that families randomly assigned to EHS were more likely to use the home visitor as a source of instrumental support, while the comparison group reported using neighbors at a higher rate (Mckelvey et al. 2015). Ninety-six percent of parents reported their primary reasons for enrolling in home visiting programs were to improve their parenting and their child’s development (Burrell et al. in press). If there is a greater match between their expectations and subsequent experiences, one would theoretically predict greater parent satisfaction (Brown et al. 2008), leading to better retention and engagement (Staudt 2007). Together, these studies suggest a potential mismatch of needs to services in the home visiting field.

While our evaluation did not focus on outcomes, studies suggest that the more time interventionists spend on child development and parent–child interaction content, the more positive the outcomes for parents and children (Pinquart and Teubert 2010; Raikes et al. 2006; Roggman et al. 2016). This holds true even for families at higher levels of socio-demographic risk (Peterson et al. 2013). Our findings further suggest that parents who got more case management in their services were less likely to be retained, decreasing the likelihood of positive outcomes. Therefore, not only are we more likely to lose families, but we may be making our services less effective for families long-term.

In our study, home visitor reports of engagement contrasted with our findings related to retention and home visit completion. When parents screened positive for depression; home visitors negatively rated their engagement. This is not surprising given that depressive symptoms might cause parents to appear less energetic, interested, or enthusiastic. Interestingly, home visitors reported better family engagement when more time was spent on case management. Home visitors might feel helpful when they are able to offer concrete, practical support to families (Jones Harden et al. 2010), and perhaps families do show appreciation and thus given higher engagement ratings. Yet overall, our findings suggest this cycle may be counterproductive.

While there are multiple strengths of the study, there are also limitations. A potential limitation of the study is the measure of depression. While the PHQ-2 is a validated instrument, there are differing recommendations for predicting major depression. Further, the manner in which the screening is conducted can have a significant impact on the rate of positive screenings. For example, screening with the PHQ-2 conducted with mothers in pediatric settings demonstrated a significantly higher positive screening rate when the screening was conducted on paper than when done in an interview format (Olson et al. 2005). The PHQ-2 in this study was conducted in an interview format, which may increase the likelihood that parents responded in a more socially desirable way to these sensitive questions. Our analyses used the lower of the recommended thresholds. We did conduct sensitivity analyses using the higher threshold (PHQ-2 scores of 3 or higher), which resulted in similar findings.

Like findings reported in other studies using staff-reported family engagement (Boller et al. 2014; Brophy-Herb et al. 2001), home visitor ratings of engagement were high and skewed to the positive. Measuring engagement as services are being provided, as we did, allows a visit-by-visit account that can be averaged across all services or examined in windows. We provided the home visitors with training and detailed descriptions of what we wanted them to reflect upon as they rated engagement, but there was still limited variability in ratings. Perhaps because the home visit was successfully completed, home visitors were more likely to rate engagement as high.

Home visitors also estimated the percent of time spent on content, which was required to total 100%, during the overall home visit. As a result, the moderators examined are associated. Home visitors must choose the focus of home visits, spending more time in case management, for example, means there was less time to spend on child development and parent–child interaction. Further, the content covered during home visits was not verified by external observation. While we are encouraged that our findings are similar to those conducted using observational coding (Peterson et al. 2007; Roggman et al. 2008), we recommend replications of our findings using observational methods.

There is a natural association between the outcome measures included in the study. The sample retained at 6 months is contained within the sample retained at 12 months. Further, while discharge decisions are likely to differ case-by-case, overall there is evidence that individuals who are less available for home visits may be more likely to leave services (Holland et al. 2014). Our analyses controlled for family demographics that have been found related to higher attrition from services (Daro et al. 2003; Hicks et al. 2008; Peterson et al. 2013; Roggman et al. 2008). However, the association between demographic risks and involvement in services is not consistent (Ammerman et al. 2006) or even linear (Korfmacher et al. 2008). It is impossible to disentangle the direction of influence between family characteristics and services in this study. These analyses were of natural variations in EBHV in one state and do not imply causation. We recommend replication of the findings in future studies. A controlled trial of additional supports for parent–child interaction could also be conducted.

The findings from the evaluation are relevant and useful to the field. They suggest the need to remain faithful to the primary purpose of services, supporting parenting and the parent–child relationship, and ultimately child outcomes (Raikes et al. 2014). In the words of the author Stephen Covey, “The main thing is to keep the main thing the main thing.” This is particularly important when thinking about the role of home visitors. A recent study included interviews with home visitors specific to their roles in supporting parent–child interactions (Jones Harden et al. 2010). Home visitors primarily reported feeling the need to focus on meeting families’ basic needs with case management work before supporting parent–child interaction during home visits, but they also reported worries that focusing on parent–child interaction could be counterproductive for parents who were emotionally vulnerable and struggled with the “distinction between facilitating and directing parent–child interaction” (p. 375). It is vital to support home visitors in their efforts to balance the complex needs of families while also individualizing services that remain focused on the key elements of services.
