# Correction to: Keeping Our Eyes on the Prize: Focusing on Parenting Supports Depressed Parents’ Involvement in Home Visiting Services

**DOI:** 10.1007/s10995-018-2619-6

**Published:** 2018-08-22

**Authors:** Lorraine M. McKelvey, Shalese Fitzgerald, Nicola A. Conners Edge, Leanne Whiteside-Mansell

**Affiliations:** Department of Family and Preventive Medicine, College of Medicine, University of Arkansas for Medical Sciences, 4301 W. Markham St, #530, Little Rock, AR 72205-7199 USA

## Correction to: Maternal and Child Health Journal 10.1007/s10995-018-2533-y

The article “Keeping Our Eyes on the Prize: Focusing on Parenting Supports Depressed Parents’ Involvement in Home Visiting Services”, written by Lorraine M. McKelvey, Shalese Fitzgerald, Nicola A. Conners Edge and Leanne Whiteside‑Mansell, was originally published electronically on the publisher’s internet portal (currently SpringerLink) on 28 May 2018 without open access. With the author(s)’ decision to opt for Open Choice the copyright of the article changed on 18 July 2018 to © The Author(s) 2018 and the article is forthwith distributed under the terms of the Creative Commons Attribution 4.0 International License (http://creativecommons.org/licenses/by/4.0/), which permits use, duplication, adaptation, distribution and reproduction in any medium or format, as long as you give appropriate credit to the original author(s) and the source, provide a link to the Creative Commons license and indicate if changes were made.

The original article has been corrected.

